# IgG Fc Glycosylation Patterns of Preterm Infants Differ With Gestational Age

**DOI:** 10.3389/fimmu.2018.03166

**Published:** 2019-01-18

**Authors:** Nele Twisselmann, Yannic C. Bartsch, Julia Pagel, Christian Wieg, Annika Hartz, Marc Ehlers, Christoph Härtel

**Affiliations:** ^1^Department of Pediatrics, University of Lübeck and University Medical Center Schleswig-Holstein, Lübeck, Germany; ^2^Laboratories of Immunology and Antibody Glycan Analysis, Institute for Nutrition Medicine, University of Lübeck and University Medical Center Schleswig-Holstein, Lübeck, Germany; ^3^Department of Infectious Diseases and Microbiology, University of Lübeck and University Medical Center Schleswig-Holstein, Lübeck, Germany; ^4^Department of Neonatology, Hospital Aschaffenburg-Alzenau, Aschaffenburg, Germany; ^5^Airway Research Center North (ARCN), German Center for Lung Research (DZL), University of Lübeck, Lübeck, Germany

**Keywords:** newborn, trans-placental transfer, IgG antibodies, IgG Fc glycosylation, mothers, galactosylation, sialylation, preterm infants

## Abstract

Preterm infants acquire reduced amounts of Immunoglobulin G (IgG) via trans-placental transfer as compared to term infants which might explain their high susceptibility for infections. The reduced amount of IgG antibodies also results in a lower amount of anti-inflammatory Fc N-galactosylated and -sialylated IgG antibodies. This reduction or, even more, a qualitative shift in the type of IgG Fc glycosylation might contribute to the increased risk for sustained inflammatory diseases in preterm infants. It was the aim of our explorative study to investigate the IgG Fc glycosylation patterns in preterm infants of different gestational ages compared to term infants and mothers of preterm infants. In plasma samples of preterm infants (*n* = 38), we investigated IgG concentrations by use of ELISA. Furthermore, we analyzed IgG Fc glycosylation patterns in plasma of preterm infants (*n* = 86, 23–34 weeks of gestation), term infants (*n* = 15) and mothers from preterm infants (*n* = 41) using high performance liquid chromatography. Extremely low gestational age infants (born < 28 weeks of gestation during second trimester) had reduced IgG concentrations and decreased proportions of galactosylated (84.5 vs. 88.4%), sialylated (14.5 vs. 17.9%) and bisecting N-acetylglucosamine-containing (8.4 vs. 10.8%) IgG Fc N-linked glycans as compared to preterm infants born ≥28 weeks of gestation (during third trimester) and term infants. Increased non-galactosylated (agalactosylated, 16.9 vs. 10.6%) IgG Fc N-linked glycans were associated with the development of chronic inflammatory bronchopulmonary dysplasia (BPD). However, mothers of preterm infants born during second or third trimester of pregnancy did not show significant differences in IgG Fc glycosylation patterns. Thus, the IgG Fc glycosylation patterns of preterm infants depend on their gestational age. Although lack of bisecting N-acetylglucosamine has been associated with less inflammatory effector functions, the decreased IgG Fc galactosylation and sialylation with lower gestational age suggest a rather pro-inflammatory pattern. The difference in IgG Fc glycosylation patterns between preterm infants and mothers of preterm infants suggests a selective enrichment of IgG glyco forms in preterm infants, which might contribute to or result of the development of sustained inflammatory diseases like BPD.

## Introduction

The adaptation of women during pregnancy leads to a feto-maternal immune tolerance, which is disrupted in the settings of preterm birth. Reasons for preterm birth are often associated with rather pro-inflammatory conditions at the feto-maternal interface (e.g., infection, age, stress) (1). With a compromised and immaturely developed immune system, preterm infants are exposed to the extrauterine environment leading to a high susceptibility for infections and sustained lung inflammation, such as bronchopulmonary dysplasia (BPD) of particular very early preterm infants (2–4).

The endogenous Immunoglobulin G (IgG) production in infants starts within 1–3 months after term birth (5). Therefore, specific immune protection of infants against pathogens is provided by the active transport of IgG through the placenta probably exclusively via the neonatal Fc receptor (FcRn) (6–10). However, IgG concentrations of the fetus are largely diminished in the second trimester of pregnancy. Specifically, during week 17–22 of gestation only 5–10% of maternal IgG concentrations are transported via the placenta, which rises to 50% during week 28–32 of gestation (11). Hence, reduced IgG concentrations in preterm infants might contribute to their predisposition for infection (9).

**Figure 1:**
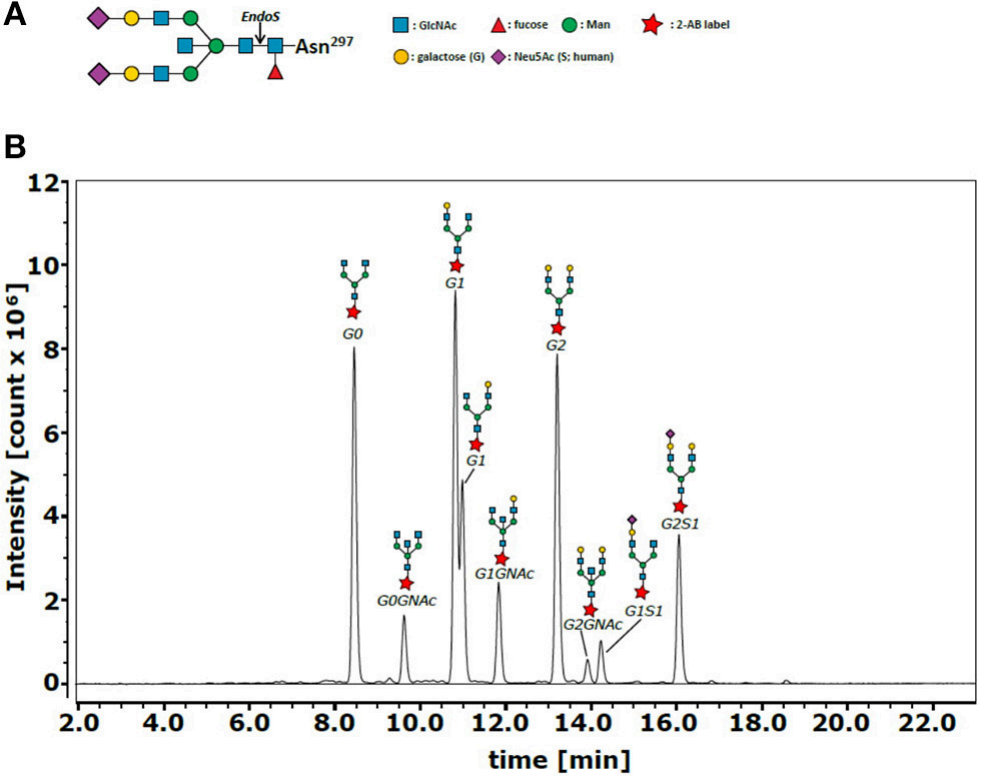
IgG Fc N-glycan structure and representative chromatogram of IgG Fc N-glycans. **(A)** The glycan structure is composed of a biantennary core heptasaccharide comprised of N-acetylglucosamines (GlcNAc or GNAc, blue) and mannoses (Man, green). The core structure can be further modified by addition of fucose (Fuc), bisecting GlcNAc (GNAc, blue), galactose (G, yellow), or sialic acid (Neu5Ac, purple). **(B)** The different Fc N-glycan modifications G0, G0GNAc, G1, G1GNAc, G2, G2GNAc, G1S1, G2S1 and G2S2 (not marked in this sample analysis) were detected using hydrophilic interaction liquid chromatography (representative chromatogram), where glycans with higher hydrophobicity (e.g., more sugar units) were retained longer by the column. Glycan composition of collected fractions containing individual peaks was previously identified by MALDI-TOF analysis (38).

In addition, a resulting reduced total amount of anti-inflammatory galactosylated and sialylated IgG antibodies (12) or, even more, qualitative changes in the type of IgG Fc glycosylation might have an impact on the specific risk profile of preterm infants including inflammation-mediated diseases. IgG Fc glycosylation patterns are characterized as follows: IgG molecules bare an N-glycosylation site on the conserved asparagine at position 297 (N297) of each heavy chain CH2 domain. The glycan is composed of a biantennary core heptasaccharide comprised of four N-acetylglucosamines (GlcNAc) and three mannoses (Figure 1A). The core structure can be further modified by addition of fucose, bisecting GlcNAc (bisection), galactose (G), or sialic acid (S) (13, 14).

IgG Fc glycosylation patterns are clinically relevant, as a shift toward more non-galactosylated (agalactosylated; G0) glycans has been linked to inflammation-mediated immune diseases (15–19). Animal models have verified that the absence or presence of galactose and sialic acid themselves can influence the inflammatory properties of IgG, i.e., pro-inflammatory [high proportion of agalactosylated glycan structures; (19, 20)] or anti-inflammatory [high proportion of galactosylated and sialylated glycan structures; (19, 21–27)]. IgG Fc glycosylation patterns without bisecting GlcNAc have been associated with reduced affinity to classical Fcγ receptors and instead less inflammatory conditions (28). The IgG Fc glycosylation pattern thereby influences not only the effector function of antigen-specific IgG in form of immune complexes, but also the immune modulatory effect of total IgG (12, 19, 21–26).

In humans, IgG Fc glycosylation patterns are variable and influenced by several factors, such as genetics, gender, age, and disease state (29–31). During pregnancy, the degree of galactosylation and sialylation of IgG antibodies increases whereas the degree of bisecting GlcNAc slightly decreases (32–36), which may contribute to the tolerance at the feto-maternal interface. Recent data from cord blood samples have confirmed that the proportion of galactosylated and sialylated IgG is higher in infants at term birth compared to older children as expected from the IgG Fc glycosylation pattern of pregnant women (37).

However, the IgG Fc glycosylation patterns of preterm infants, which are highly susceptible to infection and sustained inflammation, have not been studied yet. It was the primary objective of our explorative study to investigate the Fc glycosylation patterns in the context of preterm birth. We hypothesized that IgG Fc glycosylation patterns of preterm infants are comparable to their mother's type of IgG Fc glycosylation (feto-maternal tolerance) or are polarized toward more inflammatory properties, which might contribute to or result of the development of sepsis and sustained lung inflammation.

## Materials and Methods

### Study Cohort

We performed an explorative study in two tertiary care centers for neonates as part of our Immunoregulation of the Newborn (IRoN) study. Samples and clinical data were obtained from infants born between January 1st, 2012 and May 1st, 2015 (center 1; analyzed plasma IgG concentration of infants at mean ± SD 23 ± 2 days of life) and October 1st, 2014 and August 1st, 2017 (center 2; analyzed IgG Fc glycosylation at mean ± SD 32 ± 5 days of life). The inclusion criteria were preterm infants with gestational age ≥23.0 and ≤ 35.0 weeks without lethal abnormalities, and written informed consent provided by parents or a legal representative. At center 2 (IgG Fc glycosylation), term infants served as controls (blood withdrawal with routine newborn screening at 48–72 h of life). Form mothers of preterm infants born in center 2, we were able to obtain maternal blood from a routine sampling immediately after birth.

### Ethics

Written informed consent was obtained from parents on behalf of the infants enrolled into our studies. The study parts were approved by the local committee on research in human subjects at the University of Lübeck (center 2) and by the local committee on research in human subjects at Ärztekammer Bayern (center 1), respectively. All blood samples were obtained within a medically required blood withdrawal procedure. The additional blood volume obtained for research purposes (< 1% of whole body blood volume per blood sampling) was in line with current guidelines of the European Medical Agency on the investigation of medicinal products in term and preterm infants; Committee for Medicinal Products for Human Use and Pediatric Committee (39).

### Definitions

**Gestational age** was calculated from the best obstetric estimate based on early prenatal ultrasound and obstetric examination.

**Early-onset sepsis (EOS)** was defined as sepsis, clinical or culture-proven, occurring within the first 72 h of life.

**Late-onset sepsis (LOS)** was defined as sepsis, clinical or culture-proven, occurring after the first 72 h of life.

**Clinical sepsis** was defined as condition when neonatologists decided to treat the infant with antibiotics and continued for at least 5 days due to the following reasons: ≥2 clinical signs of systemic inflammatory response: temperature >38°C or < 36.5°C, tachycardia >200/min, new onset or increased frequency of bradycardias or apneas, hyperglycemia >140 mg/dl, base excess < -10 mval/l, changed skin color, increased oxygen need; and at least one laboratory sign: C-reactive protein >10 mg/L, platelet count < 100/nl, immature/total neutrophil ratio >0.2, white blood cell count < 5/nl (NeoKISS).

**Blood culture-confirmed sepsis** was defined as clinical sepsis with proof of causative agent in the blood culture.

**Necrotizing enterocolitis (NEC) and focal intestinal perforation (FIP)** were defined as surgery due to spontaneous intestinal perforation or necrotizing enterocolitis (Bell stage ≥2).

**Intraventricular hemorrhage (IVH)** was defined according to Papile ultrasound criteria.

**Bronchopulmonary dysplasia (BPD)** was defined as need for oxygen supplement and/or respiratory support at corrected age of 36 weeks.

**Cause of preterm delivery** was determined at the discretion of the attending obstetrician, specifically: (1) preterm labor (labor refractory to tocolytic agents) or **amniotic infection syndrome** [AIS; labor ± rupture of membranes, maternal fever (≥39.0°C), and/or one of the following signs: increased maternal inflammatory markers without any other cause (CRP > 10 mg/l or elevation of white blood cell count >15.000/μl), fetal tachycardia, painful uterus and foul-smelling cervical discharge]; (2) pre-eclampsia (pregnancy-induced maternal hypertension, oedema, proteinuria), pathological Doppler (e.g., Arteria umbilicalis Doppler, Ductus venosus flow, Arteria cerebri media Doppler), intrauterine growth restriction as diagnosed by the attending specialist for antenatal ultrasound, or placental abruption; and (3) others, including cholestasis, etc.

### Sample Collection

Peripheral blood (EDTA) samples were stored at room temperature and processed within 24 h after withdrawal. Samples were spun down for 6 min at 1,500 rpm. The clear plasma fraction was transferred and immediately stored at −80°C until further use.

### IgG ELISA

A 96 well plate was coated with anti-human IgG-Fc primary antibody (Bethyl, Laboratories, Montgomer, TX). After blocking residual binding sites, plasma dilutions were added. Intravenous immunoglobulin (IVIG, Biotech, Dreieich, Germany) was used as standard. Plasma IgG concentration was detected with horse radish peroxidase-conjugated anti-human-IgG-Fc antibody (Bethyl, Laboratories, Montgomer, TX) and assay developed with TMB substrate (BD Biosciences).

### IgG Purification and IgG-Fc Glycan Analysis

Total IgG was purified from human plasma samples using Protein G coupled agarose beads (Genetex, San Antonio, TX) in a 96 well filter plate (Merck, Darmstadt, Germany). In brief, 50 μl settled beads were constituted by washing 3 times with 200 μl of PBS and applying negative pressure on a vacuum manifold. Protein G was than incubated with 200 μl of a 1:4 plasma dilution (50 μl plasma volume) for 2 h at room temperature with agitation. Unspecific plasma proteins were washed away 5 times with 200 μl of PBS. IgG was eluted three times with 100 μl of 100 mM formic acid (pH 2.5). Elution was neutralized by adding 10 μl of 1 M ammonium bicarbonate into each elution fraction. IVIG (Biotech, Dreieich, Germany) was used as standard.

From the purified IgG, Fc N-glycan composition was analyzed as previously described (38). Briefly, Fc N-linked glycans were enzymatically released with recombinant endoglycosidase S (EndoS) from *Streptococcus pyogenes*. EndoS hydrolyses specifically the IgG Fc N-glycan of IgG after the first GlcNAc (Figure 1A). The N-glycans were purified using self-made graphitized carbon columns (Fisher Scientific, Hampton, NH) and labeled with anthranilamide (Sigma-Aldrich). The labeled glycans were analyzed by hydrophilic interaction liquid chromatography–high performance liquid chromatography (HPLC) on a Dionex Ultimate 3000 (Thermo Fischer Scientific, Waltham, Mass) by using an Xbridge XP BEH Glycan column (1.7 μm, 100 × 2.1 mm i.d.; Waters, Milford, Mass). As previously described (38), glycan composition was identified by MALDI-TOF analysis of collected fractions containing individual peaks. N-glycans with higher hydrophobicity (e.g., more sugar units) were retained longer in the column (Figure 1B).

The area under the curve (AUC) of the following nine glycan peaks were identified: G0, G0GlcNAc, G1, G1GlcNAc, G2, G2GlcNAc, G1S1, G2S1, and G2S2. Sialylated glycans with bisecting GlcNAc were not detected. The relative proportion of the individual peaks of a sample was calculated by dividing AUC of the individual curve by the AUC of the sum of all nine identified peaks and this multiplied by 100 (for raw data see Supplementary Tables 1, 2). For presentation, the four following groups were defined and percentages of the groups calculated as the sum of the percentages of the individual glycan proportions: (1) agalactosylation: G0 ± bisecting GlcNAc; (2) terminal galactosylation: G1 ± bisecting GlcNAc, G2 ± bisecting GlcNAc; (3) sialylation: G1S1, G2S1, G2S2; (4) bisection: G0 + bisecting GlcNAc, G1 + bisecting GlcNAc, G2 + bisecting GlcNAc.

### Statistical Analysis

All data were analyzed using GraphPad Prism®version 7. After testing for normal distribution, Kruskal-Wallis test followed by Dunn's multiple comparisons test or the Mann-Whitney test was used for not normally distributed data. Correlations were evaluated using Spearman correlation. Two-way ANOVA followed by Bonferroni's multiple comparison test was done for correlation with clinical parameter. The threshold for significance was a *p*-value < 0.05 depicted as ^*^, < 0.01 as ^**^, < 0.001 as ^***^, and < 0.0001 as ^****^.

## Results

### Clinical Characteristics

We recruited a cohort of preterm infants in two centers and collected plasma from peripheral blood samples and key clinical outcome parameters. In samples of center 1 we analyzed plasma IgG concentrations and in samples of center 2 we investigated IgG Fc glycosylation. To analyze whether plasma IgG concentrations and Fc glycosylation patterns differ with gestational age, we divided the cohort in 2 gestational age groups (center 1/center 2: ≥23(+0 days) and ≤ 27(+6) weeks, *n* = 19/*n* = 43; ≥28(+0) and ≤ 34(+6) weeks, *n* = 19/*n* = 43; Tables 1, 2).

**Table 1:** Summary of patient demographics from center 1 (IgG quantification).

	**<28 weeks**	**≥28 weeks**	**Total**
*n*	19	19	38
Gestational age [weeks]	26.4 ± 1.0	30.9 ± 1.9	28.7 ± 2.7
Birth weight [g]	853 ± 160	1261 ± 255	1057 ± 295
Male Gender	11 (58)	8 (42)	19 (50)
Multiple	5 (26)	6 (32)	11 (29)
EOS	0 (0)	0 (0)	0 (0)
LOS	4 (21)	4 (21)	8 (21)
NEC	0 (0)	0 (0)	0 (0)
FIP	1 (5)	0 (0)	1 (3)
IVH	4 (21)	2 (11)	6 (16)
BPD	11 (58)	3 (16)	14 (37)
**Cause of delivery**			
Preterm labor or AIS	8 (42)	5 (26)	13 (34)
Pathological doppler, placental abruption, pre-eclampsia	8 (42)	8 (42)	16 (42)
Others	3 (16)	6 (32)	9 (24)

*EOS, early-onset sepsis; LOS, late-onset sepsis; NEC, necrotizing enterocolitis; FIP, focal intestinal perforation; IVH, intraventricular hemorrhage; BPD, bronchopulmonary dysplasia; AIS, amnion infection syndrome. Data are described as mean ± standard deviation or n (%)*.

**Table 2:** Summary of patient demographics from center 2 (IgG Fc glycosylation; infants and mothers of preterm infants).

	**<28 weeks**	**≥28 weeks**	**Term infants**	**Total**
**INFANTS**				
*n*	43	43	15	101
Gestational age [weeks]	26.1 ± 1.2	29.8 ± 1.6	39.6 ± 1.2	29.7 ± 4.7
Birth weight [g]	809 ± 232	1260 ± 229	3220 ± 656	1359 ± 871
Male Gender	26 (60)	18 (42)	8 (53)	52 (51)
Multiple	11 (26)	14 (33)	2 (13)	27 (27)
EOS	13 (30)	6 (14)	1 (7)	20 (20)
LOS	22 (51)	6 (14)	0 (0)	28 (28)
NEC	2 (9)	0 (0)	0 (0)	2 (2)
FIP	4 (9)	0 (0)	0 (0)	4 (4)
IVH	12 (28)	2 (5)	0 (0)	14 (14)
BPD	14 (33)	7 (16)	0 (0)	21 (21)
**Cause of delivery**				
Preterm labor or AIS	31 (72)	27 (63)	1 (7)	59 (58)
Pathological doppler, placental abruption, pre-eclampsia	6 (14)	13 (30)	1 (7)	20 (20)
Others	6 (14)	3 (7)	13 (87)	22 (22)
**MOTHERS**				
*n*	16	25		41
Age of mother at birth [years]	33 ± 6	32 ± 4		32 ± 5
Gestational age [weeks]	25.8 ± 1.6	31.2 ± 2.1		29.1 ± 3.3
Birth weight [g]	756 ± 279	1484 ± 511		1200 ± 562
Male Gender	11 (69)	11 (44)		22 (54)
Multiple	2 (13)	4 (16)		6 (15)
EOS	7 (44)	2 (8)		9 (22)
LOS	9 (56)	1 (4)		10 (24)
NEC	2 (13)	0 (0)		2 (5)
FIP	2 (13)	0 (0)		2 (5)
IVH	4 (25)	0 (0)		4 (10)
BPD	6 (38)	0 (0)		6 (15)
**Cause of delivery**				
Preterm labor or AIS	9 (56)	14 (56)		23 (56)
Pathological doppler, placental abruption, pre-eclampsia	3 (19)	8 (32)		11 (27)
Others	4 (25)	3 (12)		7 (17)
**MOTHER-INFANT-PAIRS**				
*n*	12	8	0	20

*EOS, early-onset sepsis; LOS, late-onset sepsis; AIS, amnion infection syndrome; NEC, necrotizing enterocolitis; FIP, focal intestinal perforation; IVH, intraventricular hemorrhage; BPD, bronchopulmonary dysplasia. Data are described as mean ± standard deviation or n (%)*.

The cohort of center 1 (plasma IgG concentration) was also selected for a matched pair analysis of BPD diagnosis vs. no BPD. For this matched pair analysis early onset sepsis cases were excluded, and late onset sepsis cases were equal in both groups. In center 2, we also analyzed IgG Fc glycosylation patterns of term infants (*n* = 15) and mothers of preterm infants (*n* = 41) (Table 2). For a subgroup of preterm infants and mothers from center 2, we were able to analyze mother-infant-pairs (*n* = 20).

### Plasma IgG Concentrations Were Decreased in Preterm Infants Born at Lower Gestational Ages

**Figure 2:**
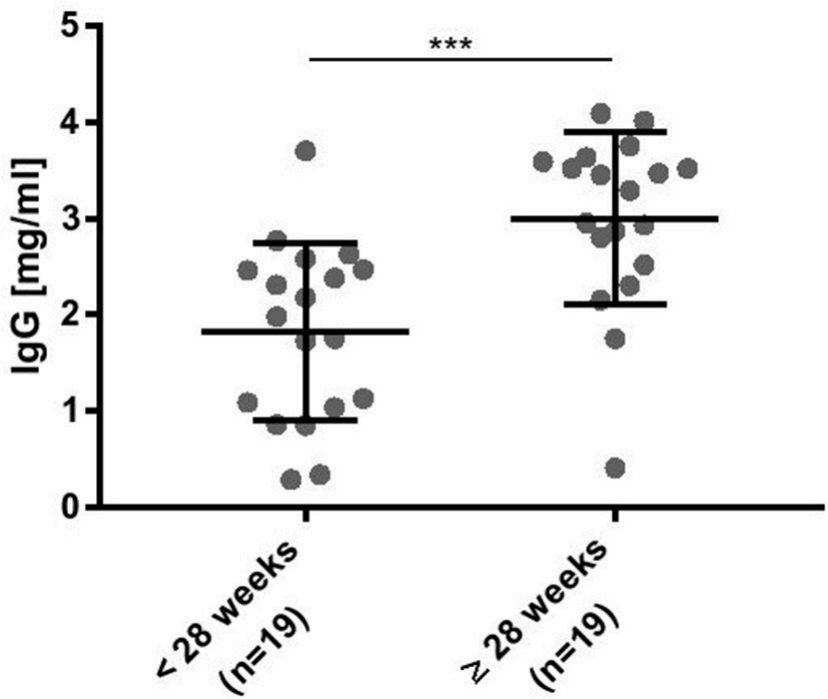
Plasma IgG concentrations were significantly decreased in preterm infants born at lower gestational ages. ELISA data for detection of human IgG-Fc parts. Intravenous immunoglobulin (IVIG) was used as a standard to estimate the plasma IgG concentration (mean ± SD, Mann-Whitney test). ^***^p < 0.001.

As outlined in Figure 2, IgG was detectable in infants born < 28 weeks of gestational age and increased significantly with gestational age. Plasma IgG concentrations were not different in a matched pair analysis of preterm infants developing BPD vs. no BPD diagnosis (data not shown).

### IgG Fc Glycosylation Patterns Depend on Gestational Age of Preterm Infants

**Figure 3:**
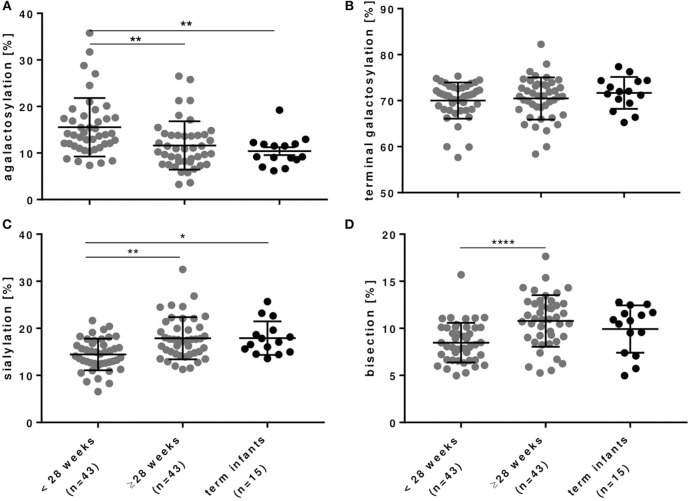
IgG Fc glycosylation in preterm infants is dependent on gestational age. **(A)** IgG with agalactosylated Fc glycans were significantly increased, whereas **(B)** IgG with terminally galactosylated Fc glycans were unchanged and IgG with **(C)** sialylated and **(D)** bisecting Fc glycans were significantly decreased in preterm infants born < 28 weeks of gestational age. Term infants served as controls (mean ± SD, Kruskal-Wallis test). ^*^p < 0.05, ^**^p < 0.01, ^***^p < 0.0001.

**Figure 4:**
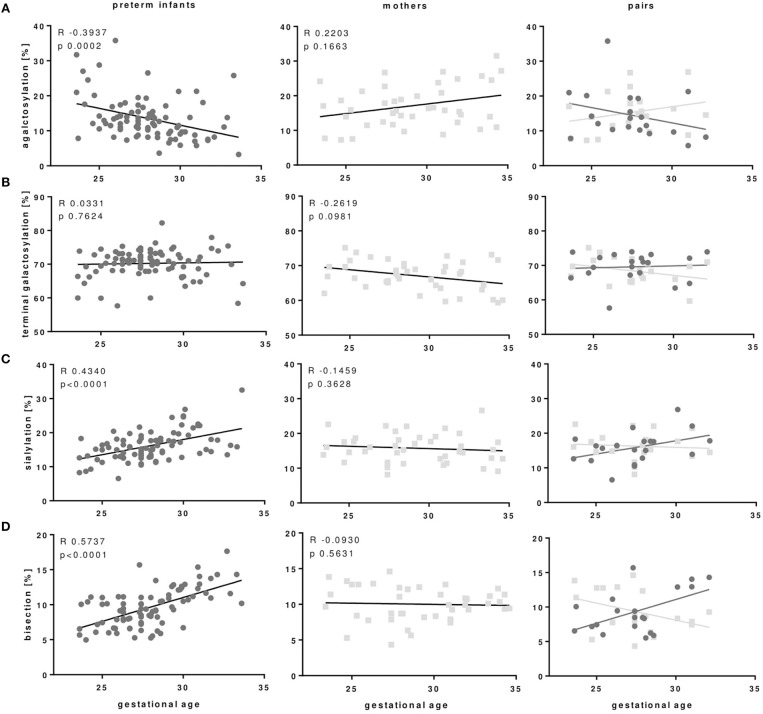
IgG Fc glycosylation patterns of preterm infants but not IgG Fc glycosylation of mothers of preterm infants correlate with gestational age of infants. **(A)** IgG with agalactosylated Fc glycans of preterm infants correlated negatively with gestational age, while Fc agalactosylation of mothers of preterm infants does not correlate with gestational age of their infants. **(C)** Sialylated and **(D)** bisecting Fc glycans correlated positively with gestational age of preterm infants which is not seen in Fc glycosylation of mothers from preterm infants. Terminal galactosylation **(B)** showed no correlation with gestational age in both preterm infants and mothers (Preterm infants: *n* = 86, mothers: *n* = 41, mother-infant-pairs: *n* = 20, Spearman correlation).

As outlined in Figures 3, 4 and Table 3, preterm infants had a higher proportion of agalactosylated (accordingly lower proportion of galactosylated) IgG Fc N-glycans with decreasing gestational age (*R* = −0.3937, *p* = 0.0002). No difference in agalactosylated IgG Fc glycans was observed between infants born ≥28 weeks of gestation and term controls. Further, no correlation between terminal galactosylation of IgG and gestational age in preterm infants was found (*R* = 0.0331, *p* = 0.76). However, also IgG Fc sialylation was significantly lower in preterm infants born < 28 weeks as compared to infants born ≥28 weeks of gestation and term infants (correlation with gestational age; *R* = 0.4340, *p* < 0.0001). The proportion of IgG glycans with bisecting GlcNAc also correlated with gestational age (*R* = 0.5737, *p* < 0.0001).

**Table 3:** IgG Fc glycosylation patterns in infants of different gestational age groups depicted in Figure 3 (mean ± SD).

	**<28 weeks**	**≥28 weeks**	**Term infants**
Agalactosylation (%)	15.5 ± 6.3	11.6 ± 5.2	10.4 ± 3.3
Galactosylation (%)	84.5 ± 6.3	88.4 ± 5.2	89.6 ± 3.3
Terminal galactosylation (%)	70.0 ± 3.9	70.5 ± 4.6	71.7 ± 3.5
Sialylation (%)	14.5 ± 3.4	17.9 ± 4.5	17.9 ± 3.6
Bisection (%)	8.4 ± 2.1	10.8 ± 2.7	9.9 ± 2.5

### IgG Fc Glycosylation Shows Differences Between Preterm Infants and Mothers of Preterm Infants

In the comparison of maternal with neonatal IgG Fc glycosylation patterns we did not find the same correlation between IgG Fc glycosylation patterns in mothers of preterm infants with the gestational age of their infants which we observed between the IgG Fc glycosylation patterns of preterm infants with their gestational age (Figure 4).

### Clinical Correlation of IgG Fc Glycosylation in Preterm Infants

For the cause of preterm delivery bisecting IgG Fc glycosylation was significantly decreased in preterm infants born ≥28 weeks of gestation with preterm labor or amnion infection syndrome (AIS) as compared to other preterm delivery reasons (Table 4).

**Table 4:** IgG Fc glycosylation patterns of preterm infants associated with (i) gender and clinical parameters defining (ii) cause of birth, (iii) bronchopulmonary dysplasia (BPD) or (iv) sepsis (early and late onset sepsis cases included) (mean ± SD, two-way ANOVA, multiple comparison within group to adjust for gestational age).

	**<28 weeks**		**≥28 weeks**	
**(i)**	**Male (*n* = 26)**	**Female (*n* = 17)**	**Male (*n* = 18)**	**Female (*n* = 25)**
Agalactosylation (%)	15.0 ± 6.5	16.3 ± 6.1	12.4 ± 6.3	11.1 ± 4.2
Galactosylation (%)	85.0 ± 6.5	83.7 ± 6.1	87.6 ± 6.3	88.9 ± 4.2
Terminal galactosylation (%)	70.3 ± 3.7	69.6 ± 4.3	70.9 ± 5.5	70.1 ± 3.8
Sialylation (%)	14.7 ± 3.5	14.1 ± 3.2	16.7 ± 4.0	18.8 ± 4.7
Bisection (%)	8.8 ± 2.3	8.0 ± 1.8	11.1 ± 3.4	10.6 ± 2.2
**(ii)**	**Preterm labor or AIS (*****n=*** **31)**	**All other causes (*****n=*** **12)**	**Preterm labor or AIS (*****n=*** **27)**	**All other causes (*****n=*** **16)**
Agalactosylation (%)	15.2 ± 5.6	16.4 ± 8.0	11.3 ± 4.0	12.2 ± 6.9
Galactosylation (%)	84.8 ± 5.6	83.6 ± 8.0	88.7 ± 4.0	87.8 ± 6.9
Terminal galactosylation (%)	70.0 ± 3.4	70.0 ± 5.3	71.2 ± 3.7	69.3 ± 5.8
Sialylation (%)	14.8 ± 3.0	13.6 ± 4.0	17.5 ± 4.1	18.6 ± 5.1
Bisection (%)	8.3 ± 1.8	9.0 ± 2.8	9.8 ± 2.3 ^***^	12.5 ± 2.7
**(iii)**	**BPD (*****n=*** **14)**	**No BPD (*****n=*** **29)**	**BPD (*****n=*** **7)**	**No BPD (*****n=*** **36)**
Agalactosylation (%)	17.0 ± 6.4	14.8 ± 6.2	16.9 ± 8.1^*^	10.6 ± 3.8
Galactosylation (%)	83.0 ± 6.4	85.2 ± 6.2	83.1 ± 8.1^*^	89.4 ± 3.8
Terminal galactosylation (%)	69.5 ± 4.2	70.3 ± 3.8	66.6 ± 5.4	71.2 ± 4.1
Sialylation (%)	13.5 ± 2.9	14.9 ± 3.5	16.5 ± 4.6	18.2 ± 4.5
Bisection (%)	8.4 ± 2.2	8.5 ± 2.1	10.9 ± 2.5	10.8 ± 2.8
**(iv)**	**Sepsis (*****n=*** **27)**	**No sepsis (*****n=*** **16)**	**Sepsis (*****n=*** **11)**	**No sepsis (*****n=*** **32)**
Agalactosylation (%)	16.6 ± 7.2	13.8 ± 3.9	14.8 ± 7.3	10.5 ± 3.8
Galactosylation (%)	83.4 ± 7.2	86.2 ± 3.9	85.2 ± 7.3	89.5 ± 3.8
Terminal galactosylation (%)	69.5 ± 4.6	70.8 ± 2.4	68.8 ± 5.5	71.1 ± 4.2
Sialylation (%)	13.9 ± 3.5	15.4 ± 3.0	16.4 ± 3.7	18.4 ± 4.7
Bisection (%)	8.7 ± 2.4	8.2 ± 1.6	12.1 ± 2.1	10.4 ± 2.8

**Figure 5:**
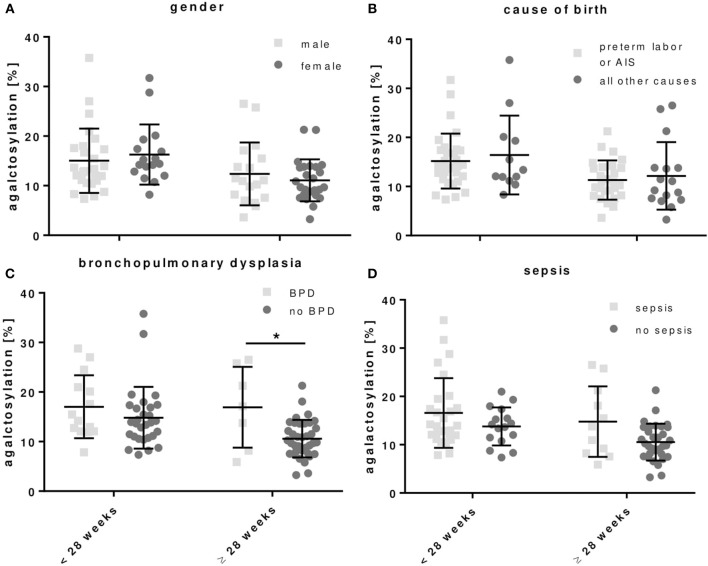
Agalactosylated IgG Fc N-glycans show a tendency to increase in preterm infants developing bronchopulmonary dysplasia (BPD). The correlation was done with (i) gender and clinical parameters defining, (ii) cause of birth, (iii) BPD, and (iv) sepsis (early and late onset sepsis cases included) for all preterm infant groups (mean ± SD, two-way ANOVA, multiple comparison within groups to adjust for gestational age). ^*^p < 0.05.

*IgG Fc agalactosylation is additionally depicted in Figure 5*.

* *p < 0.05*,

*** *p < 0.001*.

For the development of BPD, preterm infants born < or ≥28 weeks of gestation had respectively, a tending or significant increased proportion of agalactosylated IgG as compared to preterm infants without BPD (Figure 5 and Table 4). A similar tendency, which was however not significant, was observed in preterm infants with sepsis (Figure 5 and Table 4). No differences in IgG Fc glycosylation were found for the gender (Figure 5 and Table 4).

## Discussion

To our knowledge, this is the first explorative study investigating IgG Fc glycosylation patterns in peripheral blood of preterm infants. We noted that IgG Fc glycosylation patterns of preterm infants differ with gestational age whereas the patterns of mothers, as described in the literature (32, 33), did not differ with gestational age of their offspring. Preterm infants born < 28 weeks of gestation displayed an IgG Fc glycosylation pattern characterized by a higher proportion of agalactosylated IgG (reduced proportion of galactosylated IgG) and a reduced proportion of sialylated and bisecting IgG. The pattern of Fc glycosylation from mothers, however, did not differ between the second and third trimester of pregnancy suggesting an enrichment of IgG glyco forms in extremely low gestational age infants. This novel finding needs further prospective in-depth-analysis, how these IgG Fc glyco forms are enriched in the smallest, most vulnerable infants, whether they contribute to or are a result of dysregulated immune responses and sustained inflammation like BPD, and how they function.

We were able to confirm that IgG antibodies are transported through the placenta during second trimester of pregnancy, but in a much less amount in infants born < 28 weeks of gestation as compared to infants born ≥28 weeks of gestation. In our setting, IgG concentration of preterm infants ≥28 weeks of gestation were still below the levels of term born infants (clinical reference values for infants between 1 and 3 months of age are 2.5–7.5 mg/ml IgG, Immunology laboratory of the medical clinic in Freiburg, Germany, 2007).

Our current understanding of IgG Fc glycosylation patterns and its functional role for the immune system is that different Fc N-linked glycan structures can influence the inflammatory properties of IgG antibodies. Galactosylation and sialylation of total serum IgG but also of antigen-specific IgG in form of immune complexes were shown to mediate more anti-inflammatory effector functions (12, 19, 21–27) whereas a shift toward more agalactosylated and bisecting glycan structures has been associated with pro-inflammatory effects (19, 20, 28). IgG glycosylation of pregnant women in the Fc part was shown to be similar during second and third trimester of pregnancy and directed toward an anti-inflammatory pattern as compared to non-pregnant woman (32, 33). This pattern was also previously found in cord blood samples of term infants (35, 37) and was suggested to contribute to the condition of feto-maternal tolerance and permissive microbiota establishment after birth. In the context of preterm birth, however, the IgG Fc glycosylation patterns are different between infants born during second and third trimester and it is yet unknown whether immune dysregulation derives from a pro-inflammatory setting, i.e., reduced galactosylation and sialylation, or from an anti-inflammatory effect due to reduced bisection leading to lower affinities to activating Fcγ receptors. For this reason, functional studies are needed determining which IgG Fc glycosylation pattern is dominant. We showed that reduced bisection was associated with birth pathology, especially preterm labor and amnion infection syndrome (AIS) suggesting a role of Fc glycosylation patterns for preterm birth. Additionally, we showed a trend of increased IgG Fc agalactosylation in inflammatory-mediated diseases of preterm infants (i.e., chronic lung disease) supporting the hypothesis of a pro-inflammatory effect.

Since IgG of infants in the first month of life are mainly of maternal origin (5), several studies investigated the role of trans-placental transfer through FcRn to be selective for certain Fc glycosylation. *In vitro*, FcRn has a higher affinity to galactosylated IgG (40). A few studies showed a transfer of more galactosylated IgG to infants born at term by comparing IgG glycosylation in healthy pregnant women and umbilical cord blood of term infants (35, 41, 42). In another study, a similar IgG Fc glycosylation pattern between maternal sample and cord blood was reported (43). In our setting, IgG Fc glycosylation patterns in peripheral blood revealed a difference between preterm infants born during second and third trimester compared to IgG Fc glycosylation of mothers from preterm infants which is not changing over the second and third trimester. The reduced galactosylation, sialylation and bisection in extremely preterm infants might reflect the enrichment of certain IgG glyco forms at lower gestation. Since endogenous IgG production slowly starts at 1–3 months (5), we assumed that detected IgG in preterm infants is still of maternal origin. Several mechanisms for the enrichment of the described IgG glyco forms in preterm infants born < 28 weeks of gestation might be possible. First, a selective IgG transport via the placenta might play a role in early stages of pregnancy. Second, trimming (44) or extracellular modification (45) of the sugars from IgG Fc glycans in the circulation of early preterm infants might result in a dominance of the detected IgG Fc glycosylation pattern. Third, IgG in breast milk might modify the IgG Fc glycosylation pattern also it only contains about 0.01–0.06 mg/ml in women from western countries (46).

Taken together, our findings suggest an enrichment of certain IgG glyco forms in extremely low gestational age infants and that a high proportion of “pro-inflammatory” agalactosylated IgG Fc N-glycans in those infants might contribute to or result from inflammation-mediated diseases. However, IgG Fc agalactosylation was not significantly associated with the diagnosis of BPD or sepsis in extremely preterm infants, only with the diagnosis of BPD in preterm infants born ≥28 weeks of gestation. Future studies need to evaluate larger cohorts using multivariable linear regression accounting for possible confounding factors. Furthermore, functional *in vitro* and *in vivo* studies are needed to reveal consequences for effector functions of IgG glycosylation pattern in term and preterm infants.

The use of peripheral blood from infants in contrast to umbilical cord blood has the advantage not to be contaminated with maternal blood in the context of clinical sampling. Despite the limited sample volumes, we obtained peripheral blood samples of highly vulnerable infants. Our data are hypothesis-generating and add another component to the discussion of intravenous substitution of pooled immunoglobulins (IVIg) to preterm infants. Large trials have shown no benefit (INIS trial) with regard to sepsis risk or neurodevelopmental outcome (47), but to e.g., reduction of the inflammatory cytokine IL-6 (48). However, the role of IgG Fc glycosylation has never been reflected in the context of IVIg treatment in preterm infants and also not in the context of BPD. The effect might be beneficial in sustained inflammation of preterm infants, if the intravenous immunoglobulins would be immunomodulatory with higher proportions of galactosylation and sialylation, since this would simulate more closely the measured IgG glycosylation patterns in term infants and might mediate anti-inflammatory effects in preterm infants.

In summary, our data suggest that IgG Fc glycosylation patterns differ in preterm infants and their mothers suggesting an enrichment of certain IgG glyco forms in early stages of gestation. Further investigations with a larger cohort are needed to determine the enrichment and functional role of the observed IgG Fc glycosylation pattern in early preterm infants and to verify its correlation with sustained inflammation of preterm infants.

## Author Contributions

NT, YB, JP, AH, ME, and CH contributed conception and design of the study. CH and CW provided clinical samples. YB performed experiments and HPLC analysis. NT correlated HPLC analysis with clinical data and performed statistical analysis. NT and YB wrote first draft of the manuscript. All authors contributed to manuscript revision, read and approved the submitted version.

### Conflict of Interest Statement

The authors declare that the research was conducted in the absence of any commercial or financial relationships that could be construed as a potential conflict of interest.
