# Supplementary material for: IgG Fc Glycosylation Patterns of Preterm Infants Differ With Gestational Age
Source: Front Immunol. 2019 Jan 18;9:3166. doi: 10.3389/fimmu.2018.03166 (PMC6346593; doi:10.3389/fimmu.2018.03166)
Supplement: Supplementary file 1 [file Data_Sheet_1.PDF]

Supplementary Material

IgG Fc glycosylation patterns of preterm infants differ with gestational age

Nele Twisselmann[1#*], Yannic C. Bartsch[2#], Julia Pagel[1,3], Christian Wieg[4], Annika Hartz[1], Marc Ehlers[2,5], Christoph Härtel[1]

[#] **contributed equally**

*** Correspondence:**
Nele Twisselmann
nele.twisselmann@uksh.de

[1]Department of Pediatrics, University of Lübeck and University Medical Center Schleswig-Holstein, Lübeck, Germany;
[2]Laboratories of Immunology and Antibody Glycan Analysis, Institute for Nutrition Medicine, University of Lübeck and University Medical Center Schleswig-Holstein, Lübeck, Germany;
[3]Department of Infectious Diseases and Microbiology, University of Lübeck and University Medical Center Schleswig-Holstein, Lübeck, Germany;
[4]Department of Neonatology; Hospital Aschaffenburg-Alzenau, Aschaffenburg, Germany.
[5]Airway Research Center North (ARCN), German Center for Lung Research (DZL), University of Lübeck, Lübeck, Germany

Supplementary Tables

Supplementary table 1: Percentage of the area under the curve (AUC) from nine glycan peaks depicted for all preterm and term infant samples. The relative proportion of the individual peaks of a sample was calculated by dividing AUC of the individual curve (exemplary histogram in figure 1B) by the AUC of the sum of all nine identified peaks and this multiplied by 100. The glycans of collected fractions containing individual peaks were previously identified by MALDI-TOF analysis (Epp et al. 2018).

Patient _ID	Gestational age	G0	G0GNAc	G1	G1GNAc	G2	G2GNAc	G1S1	G2S1	G2S2
651	23,6	30,6221	1,1044	38,3405	3,6019	17,0731	0,9694	1,1238	7,1648	0,0000
673	23,6	20,1618	0,8298	37,3384	4,0724	23,3362	1,6397	1,6415	10,9802	0,0000
365	23,7	6,9706	0,8730	26,6587	5,8128	38,0220	3,3799	0,7678	17,5151	0,0000
689	24	26,3610	0,6715	40,0843	3,0808	19,9354	1,2233	1,3592	7,2846	0,0000
172	24,1	15,3478	2,2273	35,3795	6,3509	25,6753	1,8330	1,1969	11,9892	0,0000
521	24,3	21,1797	3,3365	35,6107	5,8032	22,8429	1,9529	1,2376	8,0365	0,0000
159	24,6	12,0803	0,0000	35,2931	3,9524	31,3950	2,1271	1,3708	13,7813	0,0000
154	24,7	19,0520	1,0592	35,9492	4,5450	25,7499	1,5710	1,4596	10,6140	0,0000
322	25	13,3495	0,8592	33,5178	4,6695	29,3536	1,9132	1,5177	14,8195	0,0000
623	25	9,8715	0,6827	31,2260	5,8968	33,8882	3,4824	0,8959	14,0564	0,0000
1032	25	24,6063	4,1935	32,6794	5,6246	20,4001	1,2140	1,4134	9,8687	n.a.
665	25,3	12,2615	0,6816	32,0762	6,7780	28,7382	2,6803	1,5696	15,2146	0,0000
572	25,4	11,7000	0,3831	34,4861	3,3996	32,1853	2,2081	1,3777	14,2602	0,0000
198	25,7	11,0327	0,4348	31,9212	4,0985	29,9808	2,1238	1,7493	18,6591	0,0000
268	25,8	16,1964	0,6872	35,2694	4,0212	28,9880	1,8976	1,2370	11,7031	0,0000
539	25,9	12,4830	0,6117	30,9028	4,0976	33,0116	2,2664	1,0551	15,5719	0,0000
539	25,9	12,4257	1,5643	31,1302	5,7644	32,6552	2,7889	0,8056	12,8657	0,0000
539	25,9	12,4267	0,6056	31,7467	3,6589	34,6157	2,2665	0,7869	13,8930	0,0000
685	25,9	7,9497	0,2340	30,8756	3,4547	37,1603	2,2221	0,8883	17,2153	0,0000
315	26	31,2238	4,5697	37,6811	5,7480	13,4142	0,8206	1,4698	5,0728	n.a.

Patient _ID	Gestational age	G0	G0GNAc	G1	G1GNAc	G2	G2GNAc	G1S1	G2S1	G2S2
681	26,1	12,7609	1,0932	33,9190	5,6428	31,1486	2,4118	0,9621	12,0618	0,0000
143	26,3	11,0030	0,0000	31,8790	5,2367	31,3986	2,7514	1,3923	16,3391	0,0000
144	26,3	16,1120	1,3168	36,0616	4,9719	26,3398	2,6365	1,1360	11,4256	0,0000
208	26,3	9,8520	0,5284	31,2328	5,6896	32,9986	3,2324	1,2822	15,1839	0,0000
212	26,3	12,2165	1,9612	30,5839	6,5875	28,3389	2,5831	1,3092	16,4197	0,0000
637	26,3	12,3577	2,0119	34,8820	6,6335	27,8819	2,3298	1,1641	12,7391	0,0000
711	26,3	11,4181	0,5888	31,4977	4,6136	36,6179	2,5807	0,7592	11,9241	0,0000
746	26,6	16,9334	1,0428	36,7732	5,0757	27,3320	1,8416	0,9334	10,0680	0,0000
568	26,7	6,9071	0,4487	29,0315	3,7248	37,6797	2,4498	0,8756	18,8828	0,0000
773	26,7	12,1646	0,5701	37,0014	4,0454	28,3597	1,9657	1,5986	14,2945	0,0000
775	26,7	13,0713	0,8908	37,6879	5,2059	25,5009	1,9608	1,6260	14,0565	0,0000
176	27	15,2436	0,0000	35,9547	3,5644	29,9188	1,7065	1,4520	12,1600	0,0000
137	27,3	7,4826	0,8443	28,4960	5,5422	37,6854	2,5356	0,7983	16,6155	0,0000
179	27,3	8,3004	2,8490	25,0820	9,2879	29,2730	3,5617	1,9636	19,4456	0,2369
296	27,4	7,5771	1,1757	28,1777	5,1469	35,0436	3,7702	1,1311	17,9776	0,0000
297	27,4	15,0309	0,4318	36,1268	4,8237	28,8716	2,1837	0,0000	12,5317	0,0000
346	27,4	13,1503	0,4890	35,9422	4,5917	28,4580	2,1368	1,3876	13,8444	0,0000
544	27,4	14,3138	1,1778	36,8754	5,6711	28,8922	2,5572	1,0864	9,4261	0,0000
560	27,4	18,1697	1,3213	37,2286	5,4005	25,2190	1,7681	1,2044	9,6884	0,0000
657	27,4	11,9389	0,0000	35,9920	6,8031	27,7723	4,2442	1,1106	12,1390	0,0000
677	27,4	12,8624	0,0000	34,0721	3,9483	32,1894	2,3689	1,3285	13,2304	0,0000
709	27,4	15,7314	1,6120	34,4890	6,4152	25,9063	2,0748	1,3891	12,3823	0,0000
761	27,4	9,4171	1,3495	29,5700	4,6523	34,4594	2,2510	1,3557	16,9451	0,0000
390	27,7	15,1519	1,5435	30,5247	5,4021	29,5497	2,4214	1,1862	14,2204	0,0000
668	27,9	18,1537	1,1723	34,9646	5,3406	25,5963	1,9693	1,2403	11,5628	0,0000
138	28	8,1936	0,0000	31,6335	5,6720	31,3693	2,9276	1,3905	18,8135	0,0000
139	28	10,2218	0,6717	36,1659	5,4751	30,3183	2,3754	0,9297	13,8421	0,0000

Patient _ID	Gestational age	G0	G0GNAc	G1	G1GNAc	G2	G2GNAc	G1S1	G2S1	G2S2
139	28	7,4154	0,3067	31,3608	4,0743	34,2215	2,4018	1,2190	18,7652	0,2355
531	28	9,5997	0,6462	32,8104	5,3209	31,5994	2,3738	1,1532	16,3159	0,1805
579	28	13,2376	0,3898	37,8412	3,0506	30,7416	1,8152	1,0012	11,9228	0,0000
1006	28	22,8640	3,6596	32,8589	5,9555	19,7596	1,4152	1,9860	11,3951	0,1061
559	28,1	13,4138	1,4089	36,2228	5,4110	25,9196	2,2044	1,5296	13,8899	0,0000
652	28,1	13,5192	0,4208	33,3197	3,1032	32,5914	2,0030	1,0506	13,9921	0,0000
122	28,3	12,4193	1,7728	31,8477	5,1326	28,8022	2,3159	1,2361	16,4733	0,0000
129	28,3	12,1409	0,0000	31,5346	6,0419	35,6439	3,0823	0,7104	10,8460	0,0000
130	28,3	12,9807	2,5083	30,9664	7,4463	31,3135	3,4962	0,8326	10,4560	0,0000
538	28,4	11,5169	0,0000	34,1373	3,7593	30,4326	2,4785	1,4867	16,1888	0,0000
764	28,4	13,1491	0,4862	34,4174	5,1478	27,9885	2,6065	1,2481	14,9563	0,0000
376	28,6	6,9216	1,1595	26,5535	5,5972	37,7367	3,6971	1,0411	17,2934	0,0000
388	28,6	9,2182	0,0000	33,5274	3,6978	33,7701	2,1910	1,2763	16,3192	0,0000
380	28,7	11,0221	1,6622	33,0349	6,3151	30,8394	2,5342	1,2393	13,3528	0,0000
609	28,7	3,5115	0,1085	25,9326	4,0949	49,0233	3,1907	0,5156	13,6229	0,0000
627	29,1	12,0250	1,7684	30,3741	5,7944	29,0581	3,3383	1,7214	15,9204	0,0000
1027	29,1	14,8123	2,2908	35,4309	7,2775	26,3856	1,8211	0,9719	10,9362	0,0736
308	29,3	8,0406	1,1787	30,0099	7,0616	33,0908	4,3597	1,6405	14,6182	0,0000
383	29,3	5,3774	1,1619	24,3480	5,2731	36,1040	3,2392	1,5189	22,9776	0,0000
136	29,4	8,2696	1,5915	30,2240	4,8340	32,2118	2,8707	1,7321	18,2663	0,0000
328	29,4	5,8552	1,6259	28,4899	7,5012	35,3550	3,7127	1,1297	16,3303	0,0000
723	29,7	6,0205	1,5254	27,6370	7,1758	34,5833	3,4064	1,5757	18,0758	0,0000
279	29,8	9,2844	1,6753	28,3499	7,4061	33,0415	3,0719	1,0557	16,1152	0,0000
392	29,9	8,9222	2,5499	25,8306	6,8359	30,5292	3,0424	1,7215	20,5684	0,0000
1017	29,9	18,1159	3,1296	35,0509	7,6554	21,4387	1,8476	1,7342	10,8595	0,1683
700	30	5,2993	0,5757	25,0836	3,8111	38,0028	2,3231	1,3857	23,5186	0,0000
1000	30	7,5561	1,5124	26,3547	6,4193	30,9872	2,5525	1,8112	22,6044	0,2021

Patient _ID	Gestational age	G0	G0GNAc	G1	G1GNAc	G2	G2GNAc	G1S1	G2S1	G2S2
317	30,1	7,5327	2,1513	25,6623	7,8611	27,0325	2,8993	2,6723	23,9204	0,2680
664	30,3	7,8945	1,7939	29,5093	6,4037	32,6202	2,5190	1,3027	17,9567	0,0000
1010	30,6	11,3753	2,4296	28,0669	6,5109	27,9697	2,2345	1,9209	19,2414	0,2509
326	30,9	7,1014	1,6921	29,2202	7,6635	28,8034	2,9585	2,5300	20,0310	0,0000
757	30,9	5,7839	1,2180	24,5996	8,6257	32,1782	5,5265	1,1448	20,6587	0,2647
522	31	4,4077	1,3664	22,0690	6,6785	38,4798	4,9006	0,9324	21,1656	0,0000
710	31	17,0288	4,2507	29,9641	7,4517	25,0363	2,3373	1,4741	12,4570	0,0000
1042	31,4	15,2833	2,7856	33,3304	7,0237	24,6824	1,8413	1,4870	13,5663	n.a.
284	31,7	6,5853	1,0124	29,1126	8,5192	36,1126	4,2190	1,0028	13,4360	0,0000
285	31,7	5,9214	1,3612	28,3809	6,8156	36,7128	2,7838	1,2785	16,7459	0,0000
377	32,1	6,6111	1,6151	27,3095	8,1730	33,9475	4,5133	1,3260	16,5046	0,0000
537	32,7	9,1509	1,9168	29,4510	10,7455	30,2423	4,9761	0,9098	12,6076	0,0000
1007	32,9	11,3653	2,4195	34,1317	7,4522	26,6188	1,7177	1,8209	14,4739	n.a.
1003	33,3	20,9032	4,8865	31,7016	7,8611	17,2283	1,5826	2,3094	13,3147	0,2127
338	33,6	2,6173	0,6351	18,2999	5,9753	36,3795	3,5861	1,9529	29,7516	0,8024
598	37,1	8,2573	0,8072	32,1713	6,6370	32,5476	3,0129	1,0493	15,4226	0,0947
812	38,1	15,8482	3,3646	33,1935	7,2340	24,0777	1,8695	1,4031	12,9106	0,0989
1067	38,4	5,1428	1,0324	23,9195	5,7742	38,7058	2,7708	1,1861	21,4684	n.a.
831	38,6	7,5240	1,6713	28,8620	6,5747	34,8560	2,6572	1,2896	16,5652	n.a.
594	38,7	6,6906	0,3061	30,2170	4,0351	40,3901	2,7349	0,9527	14,6735	0,0000
1074	39,6	9,4311	2,0816	27,4737	6,6816	28,3093	2,7906	2,6096	20,3040	0,3184
760	39,7	7,1790	1,4055	26,4966	7,0497	37,5702	3,2257	0,9934	15,9700	0,1101
292	40	5,6051	1,0324	24,3352	5,3106	34,8446	3,1739	1,5011	23,9168	0,2803
589	40	8,9361	0,0000	29,3656	3,1563	36,3508	2,5650	1,1704	18,2513	0,2045
590	40	11,4902	1,4566	32,7423	7,0944	29,3839	2,8453	1,2660	13,7214	0,0000
592	40	11,3147	0,6460	36,1078	4,7181	29,1390	2,0271	1,6405	14,4068	0,0000
593	40	8,9837	0,2075	36,6580	2,7493	34,8731	2,0141	1,1898	13,3245	0,0000

Patient _ID	Gestational age	G0	G0GNAc	G1	G1GNAc	G2	G2GNAc	G1S1	G2S1	G2S2
378	40,1	10,4143	1,1084	31,3964	6,9350	29,2294	2,7573	1,1957	16,9635	0,0000
1068	41,4	9,4790	2,4137	26,5886	7,3892	32,5016	2,9751	1,3911	17,1033	0,1583
587	42	10,2122	2,0360	32,6663	7,6482	30,9810	2,8750	1,1741	12,4073	0,0000

Supplementary table 2: Percentage of the area under the curve (AUC) from nine glycan peaks depicted for all mother samples. The relative proportion of the individual peaks of a sample was calculated by dividing AUC of the individual curve (exemplary histogram in figure 1B) by the AUC of the sum of all nine identified peaks and this multiplied by 100. The glycans of collected fractions containing individual peaks were previously identified by MALDI-TOF analysis (Epp et al. 2018).

Mother _ID	Gestational age	G0	G0GNAc	G1	G1GNAc	G2	G2GNAc	G1S1	G2S1	G2S2
34	23,4	20,9544	3,1804	33,4141	5,2085	22,1025	1,2839	1,7481	11,9961	0,1120
189	23,6	13,0408	4,0145	30,4036	7,1428	26,6563	2,6784	1,8755	13,8969	0,2913
156	23,7	5,9209	1,7900	23,1879	6,4823	36,8893	3,1132	1,2590	21,0175	0,3399
30	24,7	15,0316	3,6290	32,6835	7,3946	27,3650	2,2315	1,2022	10,3834	0,0793
67	24,7	6,8006	0,4986	29,9517	3,1510	40,4102	1,6409	1,5324	16,0148	0,0000
28	25	11,2816	2,6597	30,5775	7,3761	30,7732	2,7472	1,5781	12,7482	0,2583
167	25,3	14,0263	1,8816	32,1269	4,7818	27,7455	1,7401	1,8688	15,6753	0,1537
176	25,4	6,1310	1,3827	29,0995	6,5217	33,3332	4,8706	2,0763	16,5850	0,0000
76	26	19,1917	2,0835	35,2556	4,3761	22,9994	1,2924	2,4816	12,1604	0,1593
149	26,3	9,1825	2,2849	29,8912	7,5686	31,9655	3,0499	1,7207	14,3367	0,0000
152	27,3	9,4054	3,0515	24,1919	7,9558	29,6040	3,6051	2,6981	19,2536	0,2346
155	27,4	22,2768	0,4347	39,1116	2,7179	22,6120	1,1947	1,8996	9,7527	0,0000
174	27,4	16,9648	0,8927	37,3807	5,1502	27,5392	1,8365	1,4937	8,7422	0,0000
177	27,4	24,8637	1,8566	40,5431	5,5686	17,6970	1,3141	1,9315	6,2254	0,0000
179	27,9	9,5503	2,1156	26,9942	6,2615	31,7942	2,6814	1,3294	19,0777	0,1957
187	27,9	12,0612	2,8848	32,4880	7,2195	26,6699	2,2381	1,7534	14,5405	0,1445
171	28	17,1914	1,2290	35,3828	5,6962	24,2205	1,7880	1,5986	12,8936	0,0000
186	28,1	14,7911	0,8416	29,6452	5,2610	28,7386	2,6061	1,6748	16,2849	0,1567
172	28,4	13,8071	0,4377	36,3962	4,1486	28,1918	1,7704	1,2405	14,0078	0,0000
493	28,4	15,7590	3,1649	32,6924	6,6108	28,1573	2,3362	1,3320	9,8518	0,0955
164	28,6	9,3420	0,3261	30,4486	3,2347	32,5352	2,0761	1,9963	19,6744	0,3667
24	29,1	11,4673	2,5609	28,7140	6,9524	30,3597	2,8960	1,6838	15,2517	0,1141
487	29,4	17,8215	2,0581	30,4296	4,4780	24,7148	1,6539	2,7813	15,8364	0,2264
72	30,1	14,5264	1,8598	33,9359	4,8942	25,5464	1,5917	2,4770	14,9490	0,2195

Mother _ID	Gestational age	G0	G0GNAc	G1	G1GNAc	G2	G2GNAc	G1S1	G2S1	G2S2
90	30,4	14,1439	2,4586	30,7072	6,5830	24,7015	2,3141	1,9004	16,9546	0,2367
185	30,7	19,9562	3,8369	33,1996	6,9472	21,3651	1,9910	1,7937	10,7996	0,1107
140	30,9	18,1413	2,7035	35,0483	5,6302	25,9804	1,6007	1,2606	9,5360	0,0990
169	31	7,6520	1,1388	25,7948	4,3959	37,6230	1,9134	1,1878	20,2943	0,0000
472	31	24,2976	2,6064	31,5221	4,0415	22,8820	1,2376	1,6620	11,5028	0,2480
154	31,1	12,6034	1,7720	32,3362	4,8577	31,5676	1,8824	1,2959	13,5907	0,0942
154	31,1	15,0138	2,1896	36,5747	6,1990	30,5567	1,6139	0,5241	7,3283	n.a.
68	31,9	10,2638	2,0541	29,7526	6,1680	29,1168	2,6274	2,2096	17,6211	0,1865
473	31,9	22,0277	2,8205	32,8062	5,3152	20,5023	1,7693	2,5913	11,9979	0,1695
181	32	12,9244	2,4666	29,3075	5,7772	30,7200	2,4939	1,7873	14,4037	0,1194
160	32,1	13,6270	0,9261	33,1679	5,7265	29,4537	2,6214	1,5974	12,8800	0,0000
110	33,3	8,6302	1,6906	24,7450	5,5461	29,9082	2,8762	2,5070	23,7464	0,3504
82	33,4	22,1171	3,5965	34,1180	5,7630	22,7985	1,8035	1,2809	8,4449	0,0778
112	33,9	11,5311	2,4165	30,1442	7,1284	33,2062	2,6589	1,0328	11,7903	0,0916
64	34	21,4714	3,0341	33,3579	5,8047	19,5325	1,4964	2,2893	12,8020	0,2119
147	34,3	28,2794	3,2446	36,8354	4,9742	16,4106	1,1455	1,7465	7,2985	0,0654
184	34,3	8,6988	2,2118	25,1910	6,3581	37,2987	2,8061	0,8720	16,4034	0,1601
190	34,6	24,3846	2,8265	35,5160	5,1717	17,9384	1,4344	2,8284	9,7554	0,1446
